# Supplementary material for: Modern slavery and the race to fish
Source: Nat Commun. 2018 Nov 7;9:4643. doi: 10.1038/s41467-018-07118-9 (PMC6220235; doi:10.1038/s41467-018-07118-9)
Supplement: Supplementary file 1 — Supplementary Information [file 41467_2018_7118_MOESM1_ESM.pdf]

Modern slavery and the race to fish.

David Tickler[1,2*], Jessica J Meeuwig[1], Katharine Bryant[2], Fiona David[2], John AH Forrest[1,2], Elise Gordon[2], Jacqueline Joudo Larsen[2], Beverly Oh[1,2], Daniel Pauly[3], Ussif R Sumaila[4], Dirk Zeller[5]

1. Marine Futures Laboratory, School of Biological Sciences, University of Western Australia, Crawley, WA, 6009, Australia
2. Walk Free Foundation, Perth, WA, 6009, Australia
3. *Sea Around Us*, Institute for the Oceans and Fisheries, University of British Columbia, Vancouver, BC, V6T 1Z4, Canada
4. Fisheries Economics Research Unit, Institute for the Oceans and Fisheries, University of British Columbia, Vancouver, BC, V6T 1Z4, Canada
5. *Sea Around Us* – Indian Ocean, School of Biological Sciences, University of Western Australia, Crawley, WA, 6009, Australia

Supplementary information

Supplementary Table 1: Comparison of generalised linear models of slavery prevalence against unreported catch, mean price of catch and the proportion of fishers in the population for the top 20 countries by landings (excluding India).

Predictors	Intercept	% unreported landed catch	Mean price $/kg	Tonnes per fisher per day	df	logLik	AICc	delta	weight
**% unreported catch + price**	**0.48**	**0.62**	**-0.21**		**4**	**9.03**	**-7.40**	**0.00**	**0.66**
price + tonnes per fisher	0.65		-0.22	-0.34	4	7.47	-4.30	3.13	0.14
price	0.62		-0.24		3	5.31	-3.10	4.28	0.08
% unreported catch	0.18	0.69			3	5.10	-2.70	4.70	0.06
% unreported catch + tonnes per fisher	0.24	0.55		-0.23	4	5.88	-1.10	6.31	0.03
tonnes per fisher	0.36			-0.40	3	3.89	-0.30	7.12	0.02
Intercept only	0.30				2	1.83	1.10	8.45	0.01

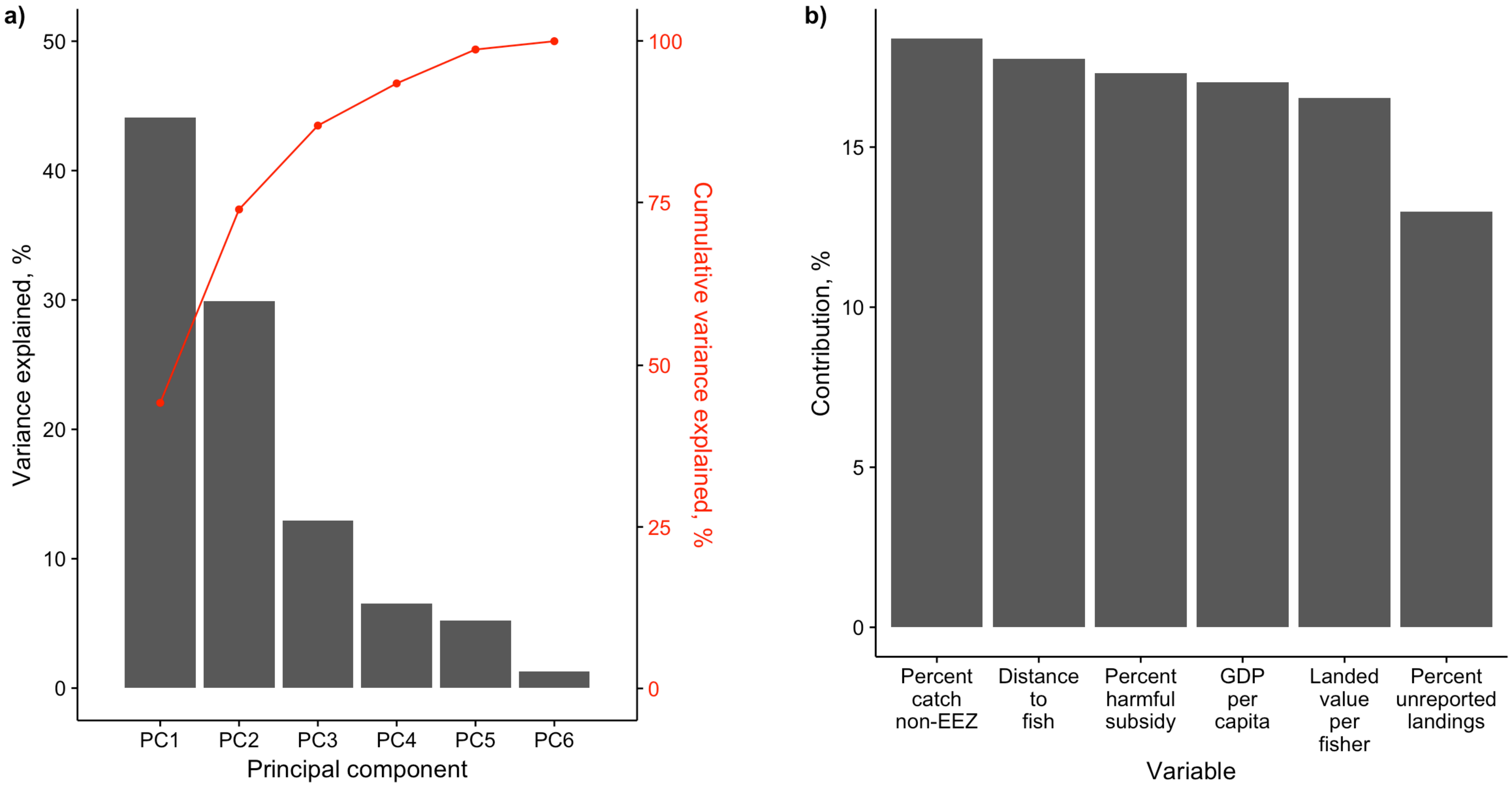

Supplementary Figure 1: a) Individual (bars) and cumulative (line) variance explained by principal components; b) Contribution of variables used in PCA to the cumulative variance explained by PC1 and PC2.

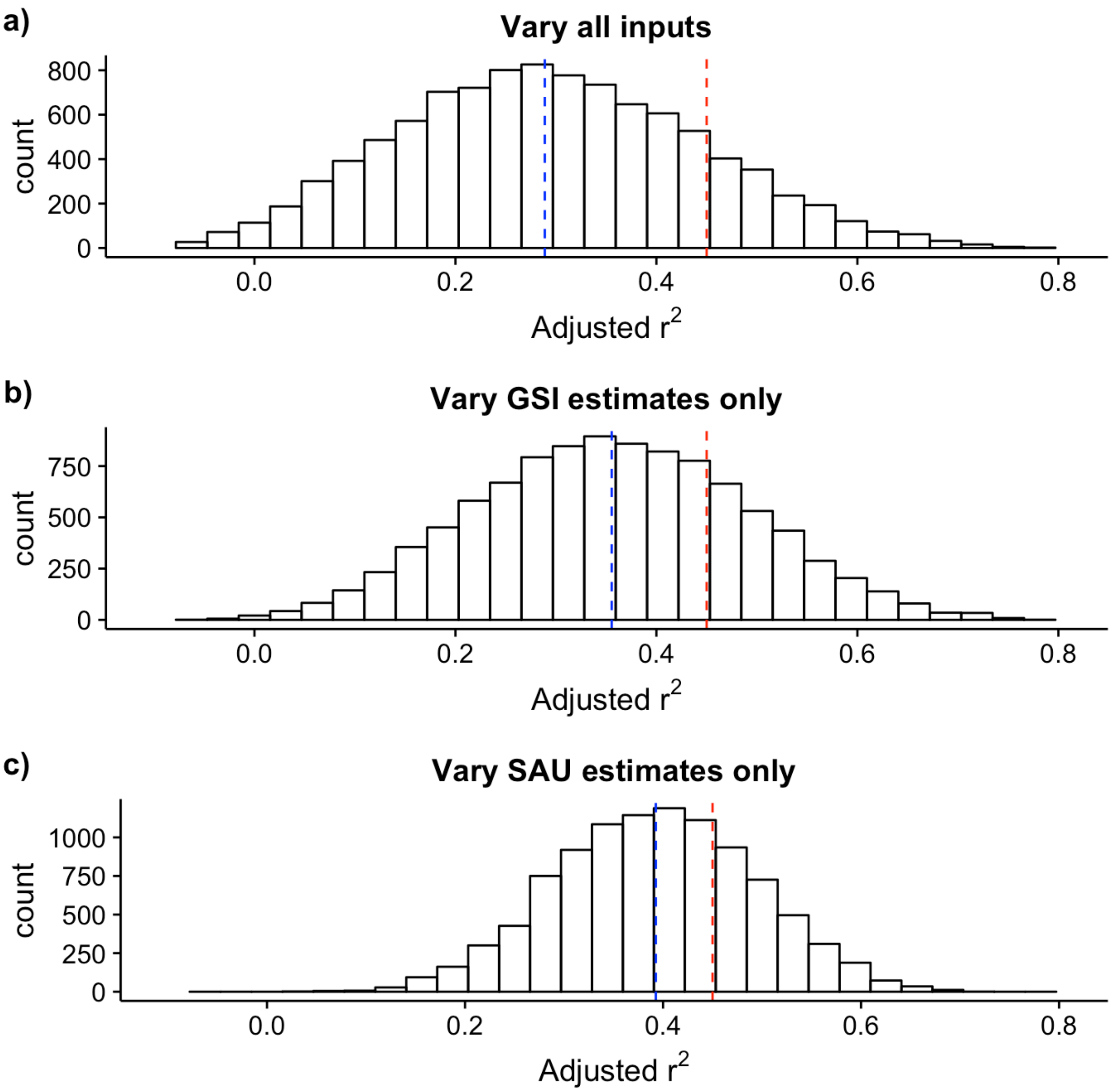

Supplementary Figure 2: Sensitivity analysis for the effect of variation in estimates for slavery prevalence, unreported catch (*IUU*) and catch price on the adjusted r^2 value for the linear model *prevalence ~ IUU + price*, for the top 20 fishing nations (excluding India):

a) varying all input variables within uncertainty range (standard deviation of Sea Around Us 10 year means; confidence interval for GSI estimates)
b) as a), but only varying the GSI estimates,
c) as a), but only varying the Sea Around Us estimates.

Dashed red lines indicate adjusted r^2 for reported model using mean or median values; dashed blue lines indicate median r^2 from 10,000 models based on resampling of input variables based on standard deviation or confidence interval.
